# Ileal hypertrophy and associated true diverticulum as a cause of colic in a horse

**DOI:** 10.4102/jsava.v88i0.1439

**Published:** 2017-05-26

**Authors:** Arnold T. Mahne, Driene Janse van Rensburg, Michael Hewetson

**Affiliations:** 1 Department of Companion Animal Clinical Studies, University of Pretoria, South Africa; 2 Department of Paraclinical Studies, University of Pretoria, South Africa

## Abstract

A 4-year-old Thoroughbred gelding underwent an explorative celiotomy for a suspected small intestinal obstruction. During surgery, an impacted diverticulum of the ileum was suspected, necessitating a jejunocaecostomy. The owner opted for euthanasia. On post-mortem examination and histopathology, a true diverticulum on the mesenteric side of the ileum, with ileal hypertrophy, was diagnosed.

## Introduction

Small intestinal diverticula occurs rarely in horses and humans compared to sheep and pigs (Cordes & Dewes 1971; Southwood et al. 2010). True diverticula are thought to be mostly congenital in origin, with Meckel’s diverticulum, seen on the antimesenteric side of the ileum, being the most commonly reported true intestinal diverticulum in the horse (Assenza et al. 2007; Southwood et al. 2010). The wall of a true diverticulum consists of all the normal histological layers (mucosa, submucosa, muscularis and serosa). False or pseudodiverticulum occurs when there is a break or rent in the muscular layer of the intestinal wall, with the mucosa and submucosa protruding through the rent. These false diverticuli are thought to be acquired (Assenza et al. 2007).

Muscular hypertrophy of the small intestine can be compensatory or idiopathic. Compensatory muscular hypertrophy occurs proximal to a chronic intestinal stenosis or obstruction and can occur in any part of the small intestine (Lindsay, Confer & Ochoa 1981). Although idiopathic muscular hypertrophy has been described in the jejunum of horses, it is mostly seen in the ileum and has been associated with diverticulum formation (Chaffin et al. 1992; Cordes & Dewes 1971). Although tapeworms (*Anaplochephala perfoliata*) have been implicated in obstruction of the ileocaecal orifice, their role in development of muscular hypertrophy of the small intestine remains undetermined (Chaffin et al. 1992).

This case report describes the clinical, surgical and pathological findings of a gelding with a true small intestinal diverticulum associated with localised ileal muscular hypertrophy.

## Case presentation

### History

A 4-year-old Thoroughbred gelding was referred to Onderstepoort Veterinary Academic Hospital for further investigation and management of colic of approximately 8-h duration. The horse had a history of recurrent colic, characterised by mild episodes of abdominal pain, which usually responded to a single dose of intravenous flunixin meglumine (Finadyne) (Intervet [Pty] Ltd., Kempton Park, South Africa).

On the day of referral, the referring veterinarian had again treated the horse for mild abdominal pain with intravenous flunixin meglumine at a dose of 1.1 mg/kg and administration of 1 g/kg of magnesium sulphate in 4 L of water via nasogastric tube. Despite an initial improvement, the horse began to demonstrate signs of severe colic 4 h later, and on follow-up rectal examination, distended loops of small intestine were palpable. At this point, the decision was made to refer the horse.

### Clinical findings

On presentation, the horse demonstrated signs of moderate abdominal pain, characterised by pawing and flank watching. Heart rate was 40 bpm with regular beat, respiratory rate was 20 bpm and temperature was 37.6 °C. Respiratory and cardiac auscultation was normal, mucous membranes pink and moist, and capillary refill time > 2 s. Abdominal auscultation revealed reduced borborygmi in all four abdominal quadrants. Digital pulses were palpable but not bounding in all four feet and the feet were cool to the touch.

On rectal examination, multiple loops of distended small intestine were palpable. Transcutaneous abdominal ultrasonography was performed with a 3.5 mHz convex transducer and revealed multiple loops of distended small intestine in the caudoventral abdomen with a diameter of up to 6 cm and poor motility. No increase in wall thickness was observed. A fluid-filled stomach was also visualised that occupied six intercostal spaces. A small quantity of anechoic free fluid was visualised in the ventral abdomen.

Three litres of gastric reflux was obtained upon nasogastric intubation. Packed cell volume was 36%, and total serum proteins were 62 g/L. On measurement of blood lactate, mild hyperlactaemia was present (4.3 mmol/L; rr < 2 mmol/L) (Arkray Lacate Pro, Carlton, NSW, Australia). Abdominocentesis yielded peritoneal fluid that was straw-coloured and clear in appearance, with a total protein of 22 g/L, a total nucleated cell count of 2 x 10 ^9^ cells/L, and a mildly elevated lactate concentration (2.4 mmol/L; rr < 2 mmol/L).

## Management and outcome

Despite intravenous administration of flunixin meglumine at 1.1 mg/kg and romifidine (Sedivet) (Boehringer Ingelheim, Randburg, South Africa) at 0.1 mg/kg, the horse became more painful and the abdomen distended, necessitating an exploratory celiotomy. Pre-operative medication included intravenous sodium benzyl penicillin (Benzyl Penicillin Fresenius) (Fresenius Kabi, Port Elizabeth, South Africa) at 30 000 iu/kg, intravenous gentamicin (Genta50) (Bayer, Isando, South Africa) at 6.6 mg/kg and a tetanus toxoid booster (Tetanus) (Onderstepoort Biological Products, Onderstepoort, South Africa) administered intramuscularly.

### Surgical findings

The horse was anaesthetised with a combination of ketamine at 2.2 mg/kg IV (Ketamine) (Fresenius Kabi, Port Elizabeth, South Africa) and diazepam at 0.1 mg/kg IV (Pax) (Fresenius Kabi, Port Elizabeth, South Africa) and maintained on isoflurane (Isofor) (Safeline Pharmaceuticals, Johannesburg, South Africa) in oxygen and constant rate infusion of ketamine at 1 mg/kg/h and medetomidine at 4 µg/kg/h (Domitor) (Zoetis [Pty] Ltd, Johannesburg, South Africa) throughout the procedure. Isotonic crystalloids (Plasmavet) (Adcock Ingram, Midrand, South Africa) were administered during general anaesthesia at 10 mL/kg/h. The gelding was placed in dorsal recumbency and the ventral abdominal area was clipped, surgically prepared and draped in a routine fashion. A standard ventral midline celiotomy was performed.

Abdominal exploration revealed multiple loops of severely distended small intestine. A 30 cm impaction of the ileum, with a firm dough-like consistency, was identified. A mass of ± 5 cm in diameter was palpable on the mesenteric ileal side, ± 15 cm orad to the ileocecal junction. The ileal impaction aborad and adjacent to the mass was cleared by gentle massage and milking into the caecum, after which the mass appeared to decrease in size and consistency. Attempts to clear the ileal impaction orad to the mass were unsuccessful: with manipulation of the proximal luminal contents, the mass would increase in size and consistency and the ileal lumen became obstructed again. An ileal diverticulum was suspected and jejunocaecostomy advised, but declined by the owner. The horse was subjected to humane euthanasia whilst under general anaesthesia.

### Pathological findings

#### Post-mortem findings

**FIGURE 1:**
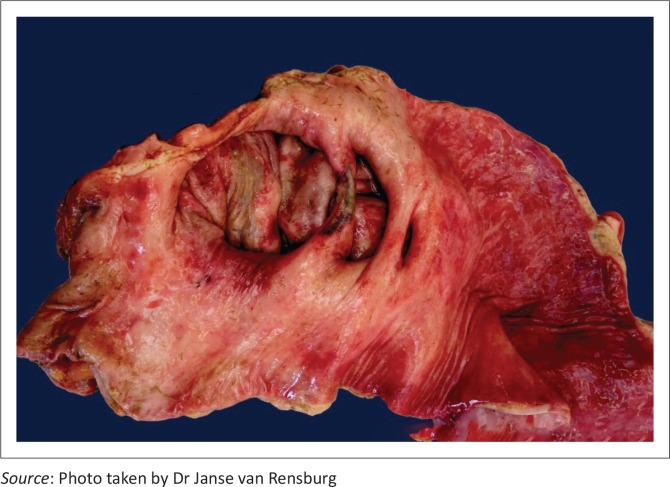
Multiple diverticula within ileal wall, immediately proximal to the ileocaecal junction.

Three locally extensive expansions within the mesenteric aspect of the ileal wall, proximal to the caecal opening, were impacted with food. These expansions were consistent with previously described diverticula (Figure 1).

#### Histopathological findings

**FIGURE 2:**
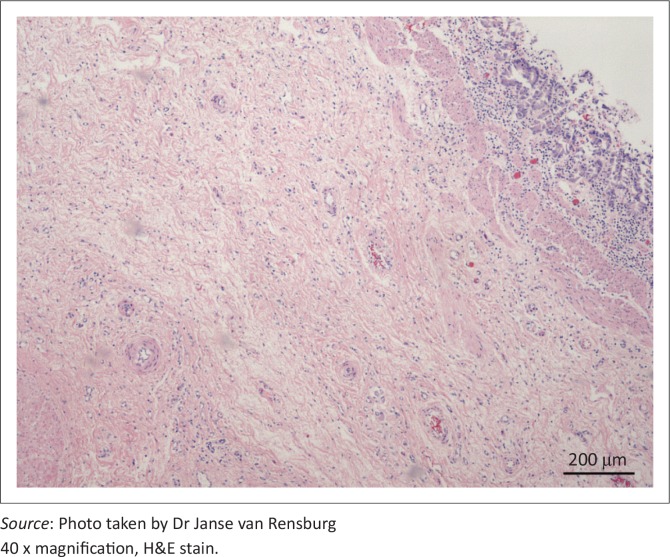
Diverticular mucosa and submucosa. The epithelial layer is thin, most likely due to *post-mortem* handling. The *lamina muscularis* is prominent and the submucosal layer mildly oedematous with infiltration of neutrophils.

**FIGURE 3:**
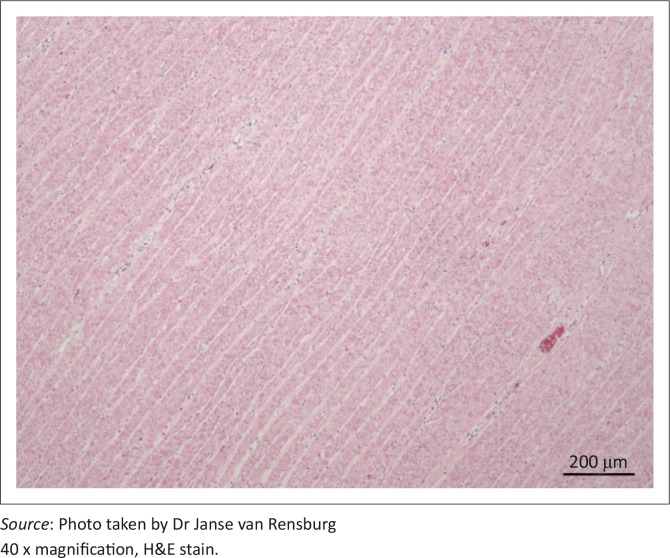
Superficial layer of severely hypertrophied circular muscular tunic.

**FIGURE 4:**
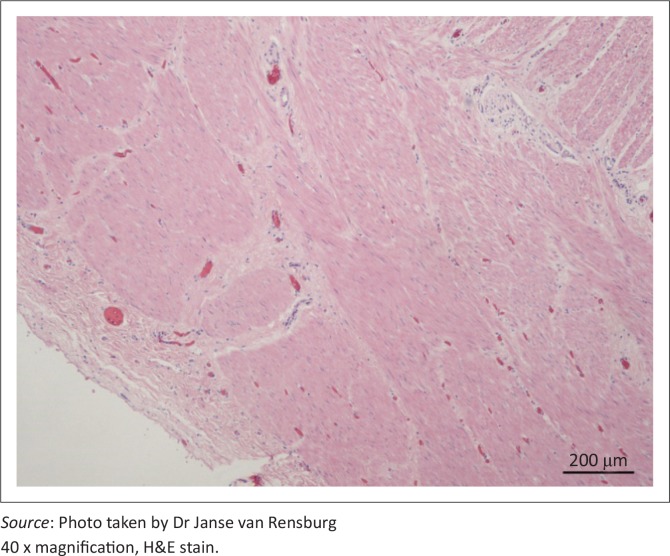
Junction between circular and longitudinal muscular tunics and serosal layer. The longitudinal muscular tunic was not as severely hypertrophied as the circular tunic.

**FIGURE 5:**
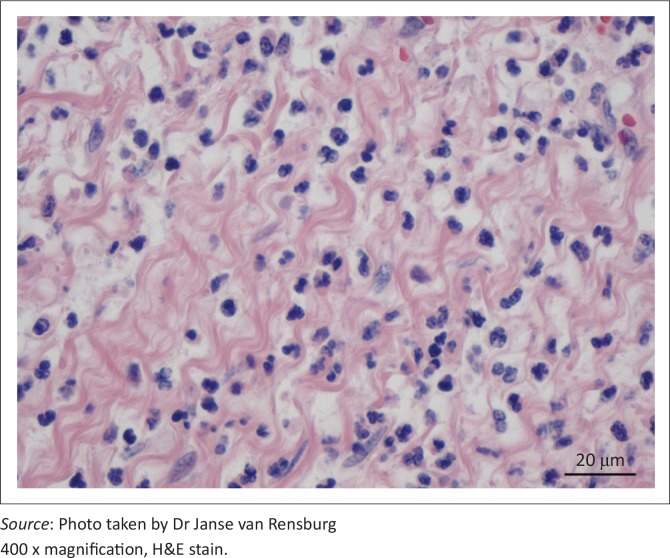
Inflammatory infiltrate, comprising neutrophils mainly within the submucosa of diverticular wall.

The diverticular wall consisted of all the normal histological layers. The mucosa was sloughed in areas due to palpation and examination of specimens. The lamina propria was slightly thickened by lymphocytic infiltration and the lamina muscularis was hypertrophied (Figure 2). The submucosa was severely thickened by fibrous connective tissue, oedema and infiltration of neutrophils. Numerous blood vessels with thickened muscular laminae, some of which contained thrombi, were also observed in the submucosa. The inner circular layer of the lamina muscularis was severely widened due to smooth muscle hypertrophy (Figures 3 and 4). The muscularis layers of the diverticular pillars were distorted by widespread necrosis and marked infiltration of neutrophils and fibrosis (Figure 5). The findings indicated chronic active enteritis.

Within the small intestine proximal to the diverticulum, multifocal areas of mucosal haemorrhage, necrosis and neutrophilic infiltration were observed. The mucosa and submucosa were severely congested and oedematous. Several blood vessels in the lamina propria, submucosa and tunica muscularis had marked hypertrophy of the tunica media. A single blood vessel lumen was occluded by a thrombus. Samples of the ileum and jejunum proximal to the diverticuli were not affected by hypertrophy of the muscular tunics.

## Discussion

Small intestinal diverticulum can be an incidental finding in horses subjected to exploratory celiotomy or at *post-mortem* examination (Cordes & Dewes 1971; Yovich & Horney 1983). However, if the diverticula become impacted, it can result in obstruction of the intestinal lumen causing colic, as was demonstrated in this case, which may necessitate surgical exploration should signs of colic not resolve with conservative management. The impaction can also cause necrosis of the diverticular wall, leading to intestinal rupture and fatal peritonitis (Weaver 1987). In addition, diverticula can result in a strangulating obstruction of the intestine or can be involved in hernias (Robertson 1990). Although impaction, peritonitis and strangulation as a result of a small intestinal diverticulum may all require surgery, the diverticulum is usually only diagnosed during surgery; however, it has been diagnosed ultrasonographically prior to surgery in a previous case report (De Solis et al. 2014).

Many authors consider all acquired diverticula to be pseudodiverticula (Assenza et al. 2007), although some subdivide acquired diverticula into true and pseudodiverticula (Robertson 1990; Southwood et al. 2010). In true acquired diverticula, increased intraluminal pressure is thought to result in protrusion of all the layers of the small intestine (Robertson 1990). This would imply that it can occur on the mesenteric or antimesenteric side of the intestine. On the contrary, pseudodiverticula occurring on the mesenteric side are thought to occur due to the penetration of the muscularis by the *vasa recta* blood vessels, resulting in weak areas in the intestinal wall, through which the mucosa and submucosa can protrude (Assenza et al. 2007).

Although the diverticulum in this report can be classified as a true diverticulum due to the presence of all the histological layers, determining the cause is problematic. Most true diverticula are thought to be congenital in origin, and reports include a large 25 cm long jejunal diverticulum in a foal, *diverticulum confluens* in an 11-year-old Trakhener horse and the well-described Meckel’s diverticulum (Riccaboni, Tassan & Mayer 2000; Verwilghen et al. 2010; Yovich & Horney 1983). Meckel’s diverticulum occurs on the antimesenteric side of the ileum and is caused by failure of the omphalomesenteric duct to regress during the first trimester of pregnancy (Hooper 1989; Verwilghen et al. 2010). Despite the fact that the diverticulum in this case is classified as a true diverticulum, it does not fit any of the published descriptions of congenital diverticula and was therefore considered to be acquired.

It is difficult to know if the ileal hypertrophy seen in this case was primary (idiopathic) or secondary (compensatory). Compensatory muscular hypertrophy of the ileum occurs as an adaptation to chronic intestinal stenosis (Chaffin et al. 1992), and it is possible that the feed-filled diverticulum in this case obstructed the intestine to such an extent that compensatory muscular hypertrophy occurred, as hypertrophied muscle is more efficient at propulsion than normal intestinal muscle (Van Kruiningen 1982, 1988). It is also possible, however, that the ileal hypertrophy seen in this case was idiopathic and that the diverticulum was caused by increased luminal pressure secondary to luminal narrowing.

Diverticula of the small intestine associated with idiopathic muscular hypertrophy are usually pseudodiverticula (Chaffin et al. 1992) (as opposed to the true diverticulum seen in this case). Although uncommon, true diverticula may, however, also be associated with idiopathic muscular hypertrophy. This has been demonstrated in a recent case report that describes muscular hypertrophy of the ileum with both true and pseudodiverticula present on the antimesenteric side (De Solis et al. 2014).

Small intestinal pseudodiverticula have also been associated with intestinal lymphoma (Mair, Pearson & Scase 2011); no evidence of this was found on histopathological examination of the ileum in this case. There was, however, evidence of chronic active inflammation and fibrosis in the diverticulum wall, presumably as a result of chronic sub-clinical impaction of the diverticulum.

A complete or partial bypass of the ileum may have been successful in this case. However, horses undergoing resection of compromised intestine during colic surgery have a decreased prognosis for survival, compared to those not requiring resection (Mair & Smith 2005). In addition, after small intestinal resection, jejunocaecal anastomosis is associated with a worse long-term survival rate, with survivors being prone to recurrent colic (Proudman, Edwards & Barnes 2007; Stewart, Southwood & Aceto 2014). Therefore, due to the decreased prognosis and financial constraints, resection and anastomosis was declined by the owner.

Peritoneal fluid analysis can help in deciding whether a colic patient needs surgery or not. Increase in total protein above 20 g/L, nucleated cell count more than 10 000 cells/µL and abnormal colour could all be indications that a horse needs abdominal exploration; however, these should not be viewed in isolation but as part of the workup. The use of peritoneal fluid lactate concentration, in this case, was to differentiate between strangulating or non-strangulating obstruction as a cause of small intestinal distention. Increased peritoneal fluid lactate concentration is a more sensitive indicator than increased plasma lactate concentration for strangulated intestine. Serosanguinous discolouration of peritoneal fluid and increased protein concentration and nucleated cell count may also be present with intestinal strangulation, depending on the duration (Freeman 2012). In the currently described case, no change in peritoneal fluid was present. The decision to perform abdominal exploration was based on distended small intestine, unresponsiveness to pain medication and progressive abdominal distention.

In conclusion, this report describes a diverticulum and muscular hypertrophy of the ileum that resulted in an ileal impaction. *Post-mortem* examination confirmed a true diverticulum on the mesenteric side of the hypertrophied ileum.
