# Corrigendum: Ileal hypertrophy and associated true diverticulum as a cause of colic in a horse

**DOI:** 10.4102/jsava.v88i0.1632

**Published:** 2017-11-30

**Authors:** Arnold T. Mahne, Drienie D. Janse van Rensburg, Michael Hewetson

**Affiliations:** 1 Department of Companion Animal Clinical Studies, University of Pretoria, South Africa; 2 Department of Paraclinical Studies, University of Pretoria, South Africa

In the version of this article initially published, Drienie D. Janse van Rensburg’s first name was misspelled as ‘Driene’ and the initial omitted. The error has been corrected in the PDF version of the article.

